# Synergistic Antibacterial Activity of Azithromycin-Loaded Chitosan Nanoparticles Alone and in Combination with Cetirizine Dihydrochloride Against Resistant Isolates of Respiratory Tract Infections

**DOI:** 10.3390/antibiotics14100992

**Published:** 2025-10-03

**Authors:** Umbreen Anwar, Adeel Sattar, Muhammad Adil Rasheed, Muhammad Abu Bakr Shabbir, Mateen Abbas

**Affiliations:** 1 Department of Pharmacology and Toxicology, Faculty of Bio-Sciences, University of Veterinary and Animal Sciences, Lahore 54000, Pakistan; umbreen100@gmail.com (U.A.); dr_aadil@uvas.edu.pk (M.A.R.); 2 Institute of Microbiology, University of Veterinary and Animal Sciences, Lahore 54000, Pakistan; abubakr.shabbir@uvas.edu.pk; 3 Quality Operations Laboratory, University of Veterinary and Animal Sciences, Lahore 54000, Pakistan; mateen.abbas@uvas.edu.pk

**Keywords:** *Klebsiella pneumoniae*, macrolide-resistant *MRSA*, AZM-CSNPs, RT-PCR

## Abstract

**Background/Objectives:** Antibiotic resistance is a major public health concern, with considerable socio-economic consequences. Researchers are exploring alternative strategies, including nanotechnology, which has shown significance in targeted drug delivery. This study evaluates the synergistic antibacterial activity of azithromycin-loaded chitosan nanoparticles (AZM-CSNPs) against azithromycin-resistant clinical respiratory isolates of *methicillin-resistant Staphylococcus aureus* (*MRSA*) and *Klebsiella pneumoniae* (*K. pneumoniae*). **Methods:** A total of 87 sputum samples (n = 87) were collected and analyzed. The *ermB* gene for *K. pneumoniae* and the *ermA* gene for *MRSA* were used to confirm resistant isolates. Among 87 samples, 29 manifested *K. pneumoniae*, and 32 exhibited MRSA-positive cultures, confirmed through phenotypic and genotypic methods. The RT-PCR is performed by using a cDNA Kit to determine the gene expression. **Results:** The results elucidate resistance of *K. pneumoniae* against several antibiotics, including azithromycin (15 µg), chloramphenicol (30 µg), and amoxicillin (30 µg), while *MRSA* also showed resistance to cefoxitin (30 µg), azithromycin (15 µg), and gentamycin (10 µg). Reduction in the MIC value of the nanoparticle formulation showed their effectiveness. The AZM-CSNPs combined with cetirizine dihydrochloride helped to down-regulate the resistant genes. **Conclusions:** Notably, a strong synergistic effect was observed with AZM-CSNPs in combination with cetirizine, significantly enhancing antibacterial efficacy against resistant isolates.

## 1. Introduction

Respiratory tract infections may be caused by different pathogens such as bacteria, viruses, or fungi. Numerous bacterial families, such as *Pseudomonas aeruginosa*, *Klebsiella pneumoniae*, *Staphylococcus aureus*, and others, have developed antibiotic resistance because of unnecessary excessive use of antibiotics [1]. Irrational use of antibiotics increases the potential for antibiotic resistance [2]. Antimicrobial resistance increases health threats on a worldwide scale. Misuse of antibiotics has resulted in the evolution of pathogenic strains that are resistant to commonly prescribed antibiotics [3,4,5]. Correct identification of pathogens and their antibiotic sensitivity testing were critical parameters for selecting suitable and successful antibiotic therapy in lower respiratory tract infections [6].

*MRSA* has a greater ability to resist most prescribed antibiotics, making it a superbug, as it has a significant role in the development of antibiotic resistance [7]. Conventional macrolides have seen scanty clinical use due to the rapid propagation of *erm* genes, which lead to macrolide resistance [8]. *MRSA* is responsible for a significant number of community- and hospital-acquired respiratory tract infections each year worldwide [9]. The two primary genes responsible for resistance in *S. aureus* are the *mecA* and *mecC* genes. These genes have been identified in phenotypically confirmed *MRSA* strains, and their resistance to various antibiotics has been reported [10,11,12].

Strong surveillance is necessary to combat the present threat of antibiotic resistance in *K. pneumoniae*, which is present in Asia [13]. *K. pneumonia* belongs to the *Enterobacteriaceae* family and is a primary cause of acquired respiratory tract infections among patients who were admitted to different wards of the hospital [14]. *K. pneumoniae* inhabits human mucosal surfaces, such as the oropharynx and gastrointestinal system. However, it can also infiltrate other tissues and result in severe infections. The World Health Organization (WHO) has identified the rising incidence of multidrug-resistant *K. pneumoniae* as a significant priority in terms of global health concerns [15]. One of the most significant challenges in the field of infectious diseases is the management of resistant *Klebsiella pneumoniae*. Despite the use of potent antibiotics, it is associated with a high prevalence of nosocomial infections with a death rate that can reach up to 50%. Consequently, to prevent the emergence of resistance, coordinated efforts addressing antibiotic stewardship and infection control are needed [16]. The majority of *K. pneumoniae* isolates, almost 84%, were categorized as multidrug resistant with high-level resistance [17]. Multiple ways have been established by *Klebsiella pneumoniae* to fight antibiotics. The activation of the efflux pump, a protein-based structure that removes undesired compounds in order to reduce their concentration within the bacterial cell, is one of the functions of the pump. It works in tandem with the decrease in membrane permeability to expel the antibiotic [18].

The semisynthetic macrolide, azithromycin, is significantly effective against both Gram-positive and Gram-negative bacteria. AZM has been effectively used to treat respiratory conditions (including asthma, bronchitis, COPD, and cystic fibrosis), enteric infections, periodontal infections, and sexually transmitted illnesses (STDs), either by itself or in combination with other antibiotics [19]. Some non-antibiotic drugs, including antihistamines, have revealed bactericidal action against different bacteria [20,21]. Cetirizine dihydrochloride, a well-known antihistamine, has exhibited antibacterial properties. The primary mechanism of action by which it expresses its antibacterial effects is by disrupting the cell membrane, thus increasing the permeability of the bacterial cell. Moreover, adherence to bacterial surfaces may increase their antimicrobial action [22,23].

New strategies, such as nanoparticles (NPs), have proved to be a promising therapeutic alternative to overcome drug resistance. These nanoscale systems have shown enhanced absorption, improved bioavailability, and higher cellular uptake of drugs [24]. NPs can infiltrate bacterial membranes and lead to disruption in molecular functions. After their combination with conventional antibiotics, they display synergistic effects that may help address the global threat of rising antibiotic resistance [25]. *K. pneumoniae*, *E. coli*, *S. aureus*, and *P. aeruginosa* were only a few of the harmful bacteria against which chitosan nanoparticles demonstrated potential antibacterial activity. In comparison to chitin and chitosan, the CS-NPs showed better antibacterial efficacy against all infections [26].

This study is designed to explore the antibacterial activity of azithromycin in combination with cetirizine dihydrochloride and azithromycin-loaded chitosan nanoparticles in combination with cetirizine dihydrochloride to overcome the resistance caused by *K. pneumoniae* and *MRSA-resistant* isolates causing respiratory tract infection. The confirmation of the targeted resistant gene in isolated samples was performed through PCR, while gene expression analysis of resistant genes of clinical isolates was studied using RT-PCR.

## 2. Results

### 2.1. Distribution of Sputum Samples

Out of a total of 87 samples, 8 (9.2%) showed no growth, while 8 (9.2%) exhibited mixed growth, 10 (11.5%) displayed fungal growth, and 61 samples (70%) contained bacterial growth.

### 2.2. Phenotypic and Genotypic Characterization of Klebsiella pneumoniae and MRSA

The bacterial samples underwent phenotypic and genotypic characterization to identify the bacterial species. The samples were first cultured on nutrient agar media and then subcultured for isolation and purification onto either a Staph-specific media or MacConkey agar. Gram staining as well as catalase, coagulase, citrate, methyl red, and VP testing were performed, and the results revealed that 32 isolates were *Staphylococcus aureus* and 29 isolates were *Klebsiella pneumoniae*. Subsequent PCR analysis showed the presence of *mecA* and *ermA* in all 32 *S. aureus* samples, thus confirming that the isolates are methicillin-resistant *S. aureus* through the following species-specific primers: *mecA* F-TCCAGATTACAACTTCACCAGG and R-CCACTTCATATCTTGTAACG and *ermA* F- TATCTTATCTTGAGAGAAGGGATT and R-CTACACTTGGCTTAGGATGAAA. Moreover, all 29 isolates of *K. pneumoniae* were shown to be positive, confirmed through the following primers: *ermB* F-CCGTTTACGAAATTTGGAACAGGTAAAGGGC and R-GAATCGAGACTTGAGTGTGC and *ermC* F-ATCTTTGAAATCGGGCTCAGG and R-CAAACCCTCTATTTGGTGGT.

### 2.3. Antimicrobial Susceptibility Testing Using Agar Well Diffusion Method for MRSA

**Figure 1:**
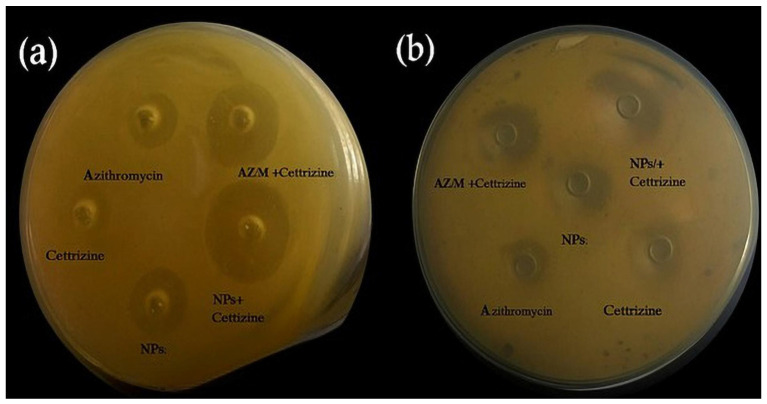
Zones of inhibition by agar well diffusion method: (**a**) *K.pneumoniae*, (**b**) *MRSA*.

The results given in Table 1 showed a significant zone diameter at the same drug concentration. The zone of inhibition was 17 mm for azithromycin against *MRSA*, while the zone diameter of AZM-CSNPs was 22 mm, and the zone diameter of cetirizine dihydrochloride was 12 mm. But when azithromycin was given in combination with cetirizine dihydrochloride, the zone diameter was 19 mm. The maximum diameter of the zone of inhibition (24 mm) was observed for the combination of nanoparticles with cetirizine dihydrochloride. An increase in zone diameter altered the resistance pattern of clinical *MRSA* isolates (n = 32); when given in an azithromycin + cetirizine dihydrochloride combination (n = 14), they turned sensitive (n = 11), intermediately sensitive, or remained resistant (n = 7). However, when the nanoparticle was given in combination with cetirizine dihydrochloride, 28 samples turned sensitive and 4 samples were intermediately sensitive. Figure 1a indicates the zone of inhibition against *MRSA.*

### 2.4. Zone of Inhibition by Agar Well Diffusion Method for Klebsiella pneumoniae

Table 2 represents the diameter of the zone of azithromycin (12 mm), cetirizine dihydrochloride (8 mm), azithromycin + cetirizine dihydrochloride (19 mm), azithromycin-loaded nanoparticles (22 mm), and azithromycin nanoparticles + cetirizine dihydrochloride (26 mm) against *K. pneumonia*. A total of n = 29 resistant *K. pneumoniae* isolates showed that all 29 resistant isolates turned sensitive when treated with a combination of nanoparticles and cetirizine dihydrochloride. Figure 1b indicates the zone of inhibition against *Klebsiella pneumoniae.*

### 2.5. Micro Broth Dilution Method

**Figure 2:**
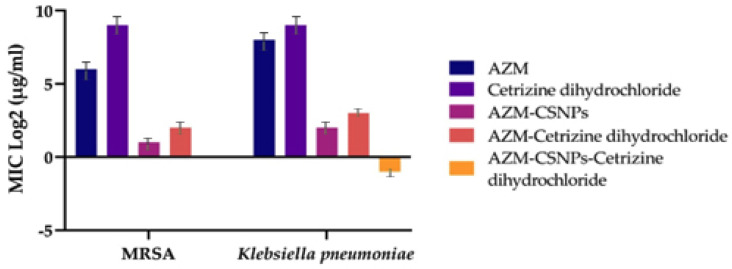
MIC (µg/mL) of isolates against different drug combinations.

Figure 2 indicates that the MIC of azithromycin 64 µg/mL, cetirizine dihydrochloride 512 µg/mL, azithromycin-loaded chitosan nanoparticle 2 µg/mL, azithromycin + cetirizine dihydrochloride 4 µg/mL, and azithromycin-loaded CSNPs in combination with cetirizine dihydrochloride was 1 µg/mL, representing a reduction in MIC against *MRSA*. Similarly, the MIC of azithromycin 256 µg/mL, cetirizine dihydrochloride 512 µg/mL, azithromycin-loaded chitosan nanoparticle 4 µg/mL, azithromycin + cetirizine dihydrochloride 8 µg/mL, and azithromycin-loaded CSNPs 0.5 µg/mL against *K. pneumoniae* revealed good results with the nanoparticle combination. Reduction in MIC value in combination and nanoparticle form explained the effectiveness of the drug combination as compared to the pure drug alone. The lower the value of MIC, the higher the efficacy.

### 2.6. Checkerboard Method

The MIC of azithromycin + cetirizine dihydrochloride against *MRSA* was 4 µg/mL, as given in Table 3, while with the AZM-CSNPs combination with cetirizine dihydrochloride, it was 1 µg/mL, as given in Table 4. There was a great reduction in MIC when given in combination. Azithromycin-loaded chitosan nanoparticles in combination with cetirizine dihydrochloride revealed greater antibacterial action. Similarly, Table 5 represents the MIC of the *K. pneumoniae* combination of azithromycin + cetirizine dihydrochloride, which was 8 µg/mL, and for AZM-CSNPs in combination with cetirizine dihydrochloride, it was 0.5 µg/mL (Table 6). The reduction in MIC was observed in combination, representing synergism. The difference in values of the combination against MRSA and *K. pneumoniae* could be due to the presence of a single peptidoglycan layer in the cell wall of Gram-negative bacteria and multiple peptidoglycan layers in Gram-positive bacteria. So, chitosan nanoparticles could be more effective against Gram-negative.

### 2.7. Gene Expression Analysis

**Figure 3:**
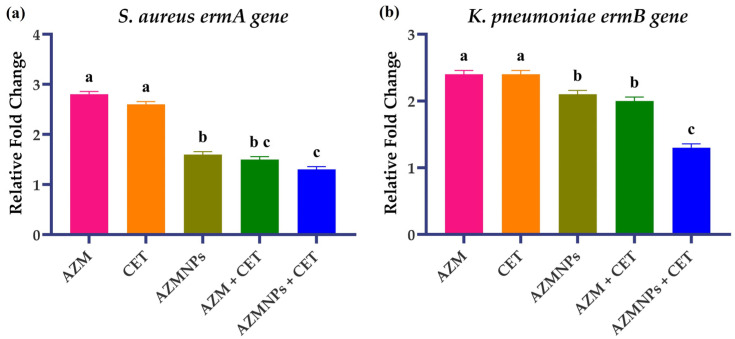
Responses of target genes, normalized using 16sRNA as a reference housekeeping gene through the Relative Expression (fold change) = 2^−ΔΔCt^ method, considering (*p* < 0.05). Note: Figure 3 explains the comparison of the relative fold expression of the *ermA* gene in *S. aureus* (**a**) and the *ermB* gene in *K. pneumoniae* (**b**). Data are expressed as mean ± SEM (n = 3). Bars annotated with different superscript letters (a, b, c) indicate statistically significant differences between groups based on multiple comparison tests (*p* < 0.05); bars sharing the same letter are not significantly different.

qRT-PCR was used to determine the association between the macrolide-resistant gene and azithromycin resistance in resistant *K. pneumoniae* and *MRSA*. The gene expression of *ermA* in *MRSA* and *ermB* in *K. pneumoniae* was observed in the presence of the housekeeping gene. There was no change, and downregulation in the expression of *ermA* and *ermB* was observed when azithromycin was given alone. Similarly, there was no effect observed in gene expression when cetirizine dihydrochloride was given alone, while there was a significant (*p* < 0.05) decrease in expression of both genes in both bacteria when treated with azithromycin + cetirizine dihydrochloride in combination. A similar decrease was observed with azithromycin-loaded chitosan nanoparticles, but there was clearly significant (*p* < 0.05) downregulation of *ermA* and *ermB* genes when AZM-CSNPs + cetirizine dihydrochloride was given in combination. So, there was an association between the azithromycin resistance due to up-regulation of *ermA* in *MRSA* and *ermB* in *K. pneumoniae* (Figure 3). Using the azithromycin-loaded chitosan nanoparticles in combination with cetirizine dihydrochloride resulted in down-regulation of resistant genes, as there was a clear decrease in relative fold change in both bacteria. Downregulation of genes represented the possibility of overcoming azithromycin resistance in respiratory tract infections caused by macrolide-resistant *MRSA* and *K. pneumoniae.*

## 3. Discussion

Respiratory tract infections (RTIs) are a substantial global source of morbidity and mortality [27]. The emergence and spread of antibacterial resistance thwart the curative power of conventional antibiotics and cause a major health crisis globally [28]. To stop the spread of MDR *K. pneumoniae*, hospital-based antibiotic stewardship programs and infection control measures must be strengthened, given the high rate of resistance [29]. *S. aureus* exhibited a significant level of resistance to commonly used antibiotics, including cefoxitin and cloxacillin, as well as ciprofloxacin, erythromycin, azithromycin, and clindamycin. *S. aureus* infections are frequently linked to high rates of morbidity, death, length of hospital stay, and financial burden [30]. This study project provided information to overcome azithromycin resistance caused by resistant respiratory isolates of *Klebsiella* and *MRSA* by targeted drug delivery through the formulation of azithromycin nanoparticles.

The phenotypic confirmation of both bacteria was performed using conventional bacteriological methods, and it was found that out of 87 samples, 29 were *K. pneumonia*, and 32 were *MRSA*. Similar findings were also observed in a study reported by [31]. Genotypic confirmation using the *mecA* gene for S. aureus (78.6%) was performed, and these findings are comparable with a study reported by [10]. Likewise, the *ermB* gene that was responsible for azithromycin resistance in *K. pneumoniae* was also amplified in this study, and this finding is consistent with the literature [32].

The antibiotic susceptibility testing of *S. aureus* and *K. pneumoniae* against different antibiotics was also performed in this study. The findings revealed that both bacteria showed high resistance against different antibiotics, including azithromycin, chloramphenicol, amoxicillin, cefoxitin, and gentamicin. Similar findings were also reported in a previous study, in which they observed a higher level of resistance to commonly prescribed antimicrobial agents [33]. Chitosan-coated polymeric nanoparticles extensively increase the antibacterial activity of tested antibiotics, including azithromycin and ciprofloxacin, against bacteria [34]. A similar trend was also reported in the current study. The results of this study represented a significant increase in the zone diameter, and the maximum diameter of the zone of inhibition was observed for AZM-CSNPs + cetirizine dihydrochloride. An increase in zone diameter altered the resistance pattern of resistant *MRSA* isolates (n = 32) and resistant *K. pneumoniae* (n = 29) isolates. All resistant isolates turned to sensitive or intermediately sensitive when treated with the combination of nanoparticles and cetirizine dihydrochloride collectively, so no sample exhibits resistance.

Gram-positive bacteria represented the improved antibacterial activity. The minimum inhibitory concentrations (MICs) of nanoparticles against the studied microorganisms were eight times lower than those of untreated azithromycin [35]. Results of our study reported the MIC of azithromycin against *MRSA* was 64 µg/mL, cetirizine dihydrochloride was 512 µg/mL, AZM-CSNPs was 2 µg/mL, azithromycin + cetirizine dihydrochloride was 4 µg/mL, and AZM-CSNPs+ cetirizine dihydrochloride was 1 µg/mL, respectively. Reduction in MIC value in combination and nanoparticle form explained the effectiveness of the drug combination as compared to the pure drug alone.

The antibacterial activity of chitosan nanoparticles (CSNPs) was projected to find widespread use in medicine as a carrier for antimicrobial medicines. The antibacterial activity of CSNPs against *K. pneumoniae, E. coli*, *S. aureus*, and *P. aeruginosa* was assessed. Analysis was also performed on the mechanism of action and variables influencing antibacterial activity, showing higher antibacterial efficacy against all pathogens [36]. In this study, the MIC value of azithromycin against *K. pneumoniae* was 256 µg/mL, that of cetirizine dihydrochloride was 512 µg/mL, that of AZM-CSNPs was 4 µg/mL, that of azithromycin + cetirizine dihydrochloride was 8 µg/mL, and that of AZM-CSNPs + cetirizine dihydrochloride was 0.5 µg/mL, respectively.

A study demonstrated a drug-loaded chitosan nanoparticle may have improved the drug’s antibacterial action and increased its penetration into the bacterial cell. Because of their antibacterial and biocompatible properties, chitosan nanoparticles were expected to find widespread use in medicine as a vehicle for antimicrobial medicines [37]. Chitosan nanoparticles strongly suppressed the development of *S. aureus* when tested for antibacterial activity; the minimum inhibitory concentration, 20 µg/mL, was lower than that of chitosan solution or amoxicillin. The antibacterial activity of amoxicillin was enhanced by complexation with chitosan nanoparticles [38]. The rational use of a fixed-dose combination of drugs may help to minimize the risk of selection of further drug resistance along with better clinical outcomes. Cefixime and azithromycin had the highest level of synergy against *K. pneumoniae* in 91% of isolates [39]. The results of our study are comparable with the above-mentioned published studies, which represent the reduction in MIC values many-fold when compared with the drug alone and in the form of nanoparticles. Most antibiotics provided enhanced effectiveness in nano-formulation or in the presence of biopolymer use in drug loading. A similar effect was observed in AZM-CSNPs that resulted in reducing MIC in both tested bacteria, but *MRSA* showed less effect compared to *K. pneumoniae*.

Chitosan nanoparticles loaded with silver were combined with azithromycin and tetracycline; synergism was observed. The additive effects were present with levofloxacin and AgNPs against *S. aureus.* Similarly, tetracycline with AgNPs against *K. pneumonia* also represented an enhanced effect [40]. A study also reported AgNPs when coupled with amoxicillin, azithromycin, clarithromycin, or linezolid, synergism was seen in 30–40% of the double combinations, and no antagonistic interactions were seen against the tested isolates [41]. The results of our study project also represented synergism when drugs were investigated in combination: azithromycin + cetirizine dihydrochloride and AZM-CSNPs + cetirizine dihydrochloride. The MIC of azithromycin + cetirizine dihydrochloride against *MRSA* was 4 µg/mL, while the AZM-CSNPs combination MIC was 1 µg/mL. There was a great reduction in MIC when the two drugs were given in combination as compared to the drug alone, and also the combination of drugs provided a synergistic effect. As described by different publishers, when two drugs are given together, they result in increased effectiveness. Also, various studies have reported that antibiotic-loaded chitosan nanoparticles have proven to be the best in efficacy by encapsulating the antibiotic with an enhanced antibacterial effect. The MIC of *Klebsiella pneumoniae* in combination with azithromycin + cetirizine dihydrochloride was 8 µg/mL, and for AZM-CSNPs it was 0.5 µg/mL; a reduction in MIC was observed in combination, representing synergism.

A study conducted by [42] compared the antibiotic alone and the antibiotic in the presence of nanoparticles. The overall findings showed that conjugating antibiotics with nanoparticles is a successful method for increasing the activity of antibiotics that have lost some of their effectiveness [43]. Our study also supports the findings of the above publisher, as an enhanced effect was observed when azithromycin was loaded on chitosan nanoparticles as compared to use alone, and a significant increase in its effectiveness was noted when studied in combination with cetirizine dihydrochloride. So, a combination of drugs provides synergistic action, and if a nanoparticle combination with a drug is used, then they produce more promising antibacterial effects and can be useful in the future to overcome resistance caused by resistant *K. pneumoniae* and *MRSA*.

A study performed by [44] for the evaluation of the efficacy of self-made tetrahedral framework nucleic acid (tFNAs) to overcome the resistance of ampicillin tFNAs was more effective in being absorbed by *MRSA* cells, as they demonstrated a higher affinity than free ampicillin for *MRSA*. The improved killing effect of tFNAs-ampicillin against *MRSA* was caused by the downregulation of genes involved in bacterial cell wall formation (murA and murZ) and the overexpression of a gene associated with antibiotic sensitivity (PBP2). Results of our study also supported the downregulation of the *ermA* gene that causes the macrolide resistance in *MRSA*. We use chitosan nanoparticles to load raw azithromycin in nanoparticles as a platform for an effective drug delivery system. A clear down-regulation of the *ermA* gene was observed when *MRSA* was treated with AZM-CSNPs in combination with cetirizine dihydrochloride, as compared to azithromycin alone, in the presence of the reference housekeeping gene in qRT-PCR. There is a downregulation of the resistant gene by treating bacteria with nanoparticles, which is helpful to overcome azithromycin resistance in clinical isolates of respiratory tract infection, specifically caused by macrolide-resistant *MRSA*.

The expression of blaOXA-48 in several clonal clusters was assessed to look into the relationship between carbapenem resistance and blaOXA-48. The level of carbapenem resistance in *K. pneumoniae* cluster A was likely correlated with the expression of blaOXA-48, suggesting that KP162’s carbapenem resistance was caused by downregulation of the respective gene [45]. The result of our study also represented the downregulation of the *ermB* gene in K. pneumoniae when bacteria were treated with AZM-CSNPs + cetirizine dihydrochloride, explaining the role of the *ermB* gene in azithromycin resistance.

Antihistaminic drugs were used in combination with the macrolide, specifically azithromycin. The observed synergistic interactions were explained by inhibition of efflux pumps and inhibition of biofilm formation. So their use as adjuvants for therapy of MDR bacterial infections mediated by overexpressed efflux pumps is promising [46]. Our study also explains that it is possible to take advantage of a synergistic combination of cetirizine dihydrochloride with azithromycin and azithromycin chitosan nanoparticles in combination with cetirizine dihydrochloride by reducing the dose of azithromycin.

## 4. Materials and Methods

### 4.1. Collection of Nanoparticles

**Figure 4:**
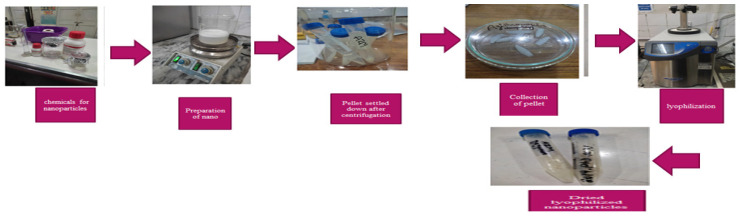
Flow sheet diagram for the ionic gelation method.

Figure 4 represents that azithromycin-loaded chitosan nanoparticles were manufactured through the ionic gelation method during the first component of the project. Chitosan solution was prepared by dissolving powdered chitosan in 1% *v*/*v* aqueous acetic acid. Tween 80 surfactant was added to the solution and then agitated at 60 °C for 2 h. The pH of the solution was adjusted to 4.4. For AZM-CSNPs, azithromycin was dissolved in CS solution (1:1 *w*/*w*). This solution was mixed with the stirring mixture at a rate of 1 mL/min in a weight ratio of 1:1 for 25 min with the addition of tripolyphosphate. Nanoparticles were collected and centrifuged at 9000 rpm for 20 min. The vials containing azithromycin-loaded chitosan nanoparticles (AZM-CSNPs) in lyophilized form were collected from the Quality Operations Laboratory, UVAS, and characterized as per standard protocol [47].

### 4.2. Sputum Samples Collection

The sputum samples from patients of all genders and age groups (*n* = 87) were collected from the pulmonary care ward of a tertiary care teaching hospital in Sialkot. Only samples with T.B.-negative cultures and showing positive growth for *MRSA* and *K. pneumoniae* were included in the study. Samples representing fungal growth, positive T.B cultures, or any other bacterial lung infections were excluded. The bacterial growth was taken directly from the Petri plates using a sterile cotton transport swab, which was then submerged in Ames transport media and labeled with the age and name of the patient. The swabs were securely packed, labeled, and shipped to the University of Veterinary and Animal Sciences in Lahore for culture and analysis. Ethical approval for this study was obtained from the Institutional Review Committee of the University of Veterinary and Animal Sciences (UVAS), Lahore, under application number 325/IRC/BMR. Informed consent was obtained from all participants before sample collection [48].

### 4.3. Phenotypic and Biochemical Identification of Clinical Isolates

The samples from the sputum were cultured by using the streak plate method on different agar media, such as blood agar or MacConkey agar, Staph 110 agar, and mannitol salt agar plates, and were incubated aerobically overnight at 37 °C. The presence of bacterial colonies was observed after 24 h of incubation. Out of 87 samples, 61 samples were studied as they showed growth of a single colony type, while a total of 26 samples were not included in the study because after culturing, they represented no growth, fungal growth, or mixed culture growth, i.e., growth showing more than one colony type of bacteria. Conventional bacteriological methods for isolation and identification include the appearance, shape, size, margins, elevations, and color of the colony. A Gram staining procedure for the separation of Gram-positive from Gram-negative bacteria and biochemical characteristics testing were used for bacterial identification. Also, specific resistant genes were targeted and observed by using PCR for confirmation of Klebsiella pneumoniae and methicillin-resistant Staphylococcus aureus [49].

### 4.4. Genotypic Confirmation of Clinical Isolates

Polymerase chain reaction (PCR) of 87 isolates was carried out for identification by using species-specific primers. Primers were diluted and vortexed for use in PCR. The specific resistant gene was targeted and observed by using PCR for confirmation of resistant isolates of *K. pneumoniae* and *MRSA*. The *ermB* gene, which causes azithromycin resistance, and the *ermC* gene, which controls efflux pump activation, were utilized to confirm *K. pneumoniae*, while *the* mecA gene and *ermA* were used for confirmation of *S. aureus*. Initial denaturation was performed for 5 min at 95 °C, followed by 30 cycles of one minute at 94 °C for denaturation and one minute at 60 °C for annealing as given in Table 7 [50]. Target genes and primer details used in the study are given in Table 8.

### 4.5. Antibiotic Susceptibility Testing

The Kirby–Bauer disk diffusion method was employed for determining the azithromycin resistance pattern of all confirmed isolates of *S. aureus* and *Klebsiella pneumoniae*. Interpretation of the zone of inhibition was performed by using the CLSI guidelines 2023 [51].

### 4.6. Evaluation of Antibacterial Activity by Agar Well Diffusion Method

In vitro, the antibacterial activity of non-antibiotics, nanoparticles, and their combinations was evaluated by using the agar well diffusion method. Stock solutions of azithromycin, cetirizine dihydrochloride, and azithromycin-loaded chitosan nanoparticles were prepared with a strength of 2000 µg in an Eppendorf. 100 µL of diluent was added to each of 10 Eppendorf tubes initially, so the first Eppendorf tube contains 1000 µg in 100 µL. 100 µL from the first Eppendorf was taken and added to the second, and the process was continued till the 10th Eppendorf tube, and two-fold serial dilutions were performed. In the other 10 Eppendorf tubes, stock solutions of azithromycin, cetirizine dihydrochloride, and azithromycin-loaded chitosan nanoparticles were prepared to obtain 1:1 (azithromycin + cetirizine dihydrochloride) and (azithromycin-loaded chitosan nanoparticles + cetirizine dihydrochloride) with the strength of 2000 µg in Eppendorf. 100 µL from the second tube containing 500 µg of azithromycin was taken and transferred to another Eppendorf containing cetirizine dihydrochloride to form the drug combination. Similarly, 100 µL from the second Eppendorf containing 500 µg azithromycin-loaded chitosan nanoparticles was transferred to the Eppendorf containing cetirizine dihydrochloride to obtain a drug combination (1:1). Each type of tested bacterial culture was grown in nutrient broth to attain 1.5×10^8^ CFU/mL and then swabbed on the surface of MH agar on Petri plates separately. A sterile borer was used to make wells (8 mm) on the agar surface. Subsequently, samples amounting to 100 µL from each dilution of the above-tested samples were collected and poured into respective wells. The Petri plates were incubated at 37 °C for 24 h, and the zone diameter (mm) around the well was measured [52].

### 4.7. Determination of Minimum Inhibitory Concentration by the Micro Broth Dilution Method

The MIC of azithromycin and AZM-CSNPs was determined against *MRSA* and *K. pneumoniae* isolates using the micro broth dilution method. Also, the tested drugs and their combination were placed in each well in a concentration range of 1000 µg/mL–0.49 µg/mL following two-fold serial dilutions up to the 12th well, while 100 µL were discarded from the 12th well. The plates were sealed tightly and incubated for 24 h at 37 °C. MIC was observed considering positive control, inoculated medium without antibiotic, and negative control medium without bacteria (only broth). MIC was estimated by visible inhibition of bacterial growth by the antimicrobial agent [53].

### 4.8. Determination of Synergistic Potential by Checkerboard Method

The potential synergistic effect of azithromycin and azithromycin nanoparticles in binary combinations was evaluated through a checkerboard assay. For this purpose, 100 µL of Muller Hinton broth was dispensed in all wells of the 96-well micro-titration plate, followed by the addition of 100 µL of azithromycin or azithromycin nanoparticles in wells of the 1st column and making two-fold serial dilutions up to the 10th well of respective rows, attaining the varying concentrations ranging from 1000 to 1.95 µg/mL, respectively. Subsequently, 100 µL of azithromycin and cetirizine dihydrochloride were added to respective wells of the 1st column and made two-fold serial dilutions vertically. A sample amounting to 50 µL from each bacterial suspension was added to all wells of a 96-well plate and incubated at 37 °C for 24 h. The fractional inhibitory concentration index (FICI) of drug combinations was determined by measuring the optical density at 600 nm [54]. To calculate the FIC index (FICI), the following formula was utilized:

FIC_A_ = MIC of A alone/MIC of A in combination.

FIC_B_ = MIC of B alone/MIC of B in combination.

FICI = FIC_A_ + FIC_B._

Where FIC_A_ = MIC of drug A in the combination/MIC of drug A alone; FIC_B_ = MIC of drug B in the combination/MIC of drug B alone. The values of FICI 0.5, >0.5–1.0, >1.0–4.0, and >4.0 are interpreted as synergistic, additive, indifferent (non-interactive), and antagonistic effects [55].

### 4.9. Identification of Gene Expression After Exposure to Various Drugs Alone and in Combination with Nanoparticles by qRT-PCR

The RT-PCR technique was used to determine the gene expression analysis of the *ermB* gene in *K. pneumoniae* and the *ermA* gene in *S. aureus*. Both respiratory-resistant isolates were exposed to drugs and their combinations. RNA was extracted using an RNA extraction kit from each sample. A cDNA Kit (Thermo Scientific, USA) was used to make cDNA. Briefly, the bacteria were cultured in Mueller-Hinton broth at 37 °C for 24 h. After incubation, RNA was finally stored at −80 °C. The reverse transcription method was used to synthesize cDNA by using the Takara cDNA synthesis kit and finally stored at 4 °C. Quantitative real-time PCR was carried out by a real-time PCR kit. Real-time PCR mixture purchased from Thermo Fisher Scientific (MA, USA), named Maxima Sybr Green/ROX qPCR Master Mix (CATALOG #K0221), was used for gene expression analysis of resistant genes. A housekeeping gene (16S rRNA) was used as an internal control for normalization. The experiment was repeated three times to calculate the mean fold change. Each reaction of real-time PCR was amplified in duplicate, and the relative fold change was determined [56].

### 4.10. Statistical Analysis

The data were compiled in Microsoft Excel (version 2019), and frequencies of sample positivity were presented as descriptive statistics (i.e., percentages). The statistical analysis was performed using one-way ANOVA followed by Tukey’s multiple range comparison. The level of significance was kept at *p* < 0.05. Analysis was performed by using GraphPad Prism version 10.3.1 (GraphPad LCC, San Diego, CA, USA) [57].

## 5. Conclusions

The findings of the present study indicated that AZM-CSNPs not only have an antibacterial effect but also represent a synergetic effect when combined with cetirizine dihydrochloride against *S. aureus* and *K. pneumonia*. Reduction in MIC_50_ was observed for both tested bacteria when given in AZM-CSNPs + cetirizine dihydrochloride as compared to azithromycin alone. Furthermore, the results proved that AZM-CSNPs + cetirizine dihydrochloride cause downregulation of azithromycin-resistant genes and thus may be helpful to overcome azithromycin resistance caused by resistant Klebsiella and MRSA in respiratory tract infections in the future. However, further in vivo studies are still required to evaluate the therapeutic safety and pharmacokinetic effectiveness of nanoparticles.

## Figures and Tables

**Table 1:** Resistance pattern of *MRSA* (n = 32). Zones of inhibition are expressed as mean values (mm) with corresponding median and interquartile range (IQR).

Sr	Sensitivity	AZM		Cetirizine		AZM+ Cetirizine		AZM-CSNPs		AZM-CSNPs + Cetirizine	
		Mean	IQR	Mean	IQR	Mean	IQR	Mean	IQR	Mean	IQR
1	Susceptible	0	0 (0–0)	0	0 (0–0)	14	14 (12–16)	18	18 (16–20)	28	28 (26–30)
2	Intermediately Susceptible	6	6 (4–8)	0	0 (0–0)	11	11 (9–13)	12	12 (10–14)	4	4 (3–5)
3	Resistant	26	26 (24–28)	32	32 (30–34)	7	7 (6–9)	2	2 (1–3)	0	0 (0–0)

The interpretive sensitivity criteria were ≥18 mm = sensitive, ≤13 mm = resistant, and 14–17 mm = intermediate.

**Table 2:** Resistance pattern of *Klebsiella pneumoniae* (n = 29).

Sr #	Sensitivity Pattern	Azithromycin	Cetirizine	Azithromycin + Cetirizine	Azithromycin NPs	Azithromycin NPs+ Cetirizine
**1**	**Susceptible**	0	0	18	21	29
**2**	**Intermediately** **Susceptible**	0	0	0	8	0
**3**	**Resistant**	29	29	11	0	0

The interpretive sensitivity criteria were ≥ 13 mm = sensitive, and ≤12 mm = resistant.

**Table 3:** Synergistic action of azithromycin (A) and cetirizine dihydrochloride (B) against *MRSA*.

MIC µg/mL (Combination)	MIC (A)	MIC (B)	FICI	Interpretation
4	1024	512	0.011	Synergism
4	512	512	0.015	Synergism
4	256	512	0.023	Synergism
4	128	512	0.039	Synergism
4	64	512	0.070	Synergism

**Table 4:** Synergistic action of AZM-CSNPs (A) and cetirizine dihydrochloride (B) against *MRSA*.

MIC µg/mL (Combination)	MIC (A)	MIC (B)	FICI	Interpretation
1	1024	512	0.0029	Synergism
1	512	512	0.0039	Synergism
1	256	512	0.0058	Synergism
1	128	512	0.0097	Synergism
1	64	512	0.0175	Synergism

**Table 5:** Synergistic action of azithromycin (A) and cetirizine dihydrochloride (B) against *K. pneumoniae*.

Sr #	MIC µg/mL (Combination)	MIC (A)	MIC (B)	FICI	Interpretation
1	8	1024	512	0.0234	Synergism
2	8	512	512	0.0312	Synergism
3	8	256	512	0.0468	Synergism

**Table 6:** Synergistic activity of AZM-CSNPs(A) and cetirizine dihydrochloride (B) against *Klebsiella pneumoniae*.

Sr #	MIC (µg/mL) Combination	MIC (A)	MIC (B)	FICI	Interpretation
1	0.5	1024	512	0.0014	Synergism
2	0.5	512	512	0.0019	Synergism
3	0.5	256	512	0.0029	Synergism

**Table 7:** Thermocycling conditions for different PCR primers.

Target Gene	Initial Denaturation	Denaturation	Annealing	Extension	Final Extension
*mecA*	94 °C/3 min	94 °C/30 s	54 °C/1 min	72 °C/20 s	72 °C/4 min
*ermA*	94 °C/3 min	94 °C/30 s	56 °C/30 s	72 °C/10 s	72 °C/4 min
*ermB*	95 °C/3 min	95 °C/30 s	57 °C/30 s	72 °C/22 s	78 °C/4 min
*ermC*	95 °C/3 min	95 °C/30 s	58 °C/30 s	72 °C/15 s	77 °C/4 min

**Table 8:** Target genes and primer.

Target Genes	Primer Sequence	Amplicon Size
*mecA* (*MRSA*)	F-TCCAGATTACAACTTCACCAGG R-CCACTTCATATCTTGTAACG	310
*ermA* (*MRSA*)	F-TATCTTATCTTGAGAAGGGATT R-CTACACTTGGCTTAGGATGAAA	139
*ermB* (*K. pneumoniae*)	F-CCGTTTACGAAATTTGGAACAGGTAAAGGGC R-GAATCGAGACTTGAGTGTGC	359
*ermC* (*K. pneumoniae*)	F-ATCTTTGAAATCGGCTCAGG R-CAAACCCTCTATTTGGTGGT	259

## Data Availability

All the data are incorporated in the manuscript file. No supplementary file is available.
